# Irisin, Two Years Later

**DOI:** 10.1155/2013/746281

**Published:** 2013-11-05

**Authors:** Marta G. Novelle, Cristina Contreras, Amparo Romero-Picó, Miguel López, Carlos Diéguez

**Affiliations:** ^1^Department of Physiology, CIMUS, University of Santiago de Compostela-Instituto de Investigación Sanitaria (IDIS), 15782 Santiago de Compostela, Spain; ^2^CIBER Fisiopatología de la Obesidad y Nutrición (CIBERobn), 15706 Santiago de Compostela, Spain

## Abstract

In January 2012, Boström and colleagues identified a new muscle tissue secreted peptide, which they named irisin, to highlight its role as a messenger that comes from skeletal muscle to other parts of the body. Irisin is a cleaved and secreted fragment of FNDC5 (also known as FRCP2 and PeP), a member of fibronectin type III repeat containing gene family. Major interest in this protein arose because of its great therapeutic potential in diabetes and perhaps also therapy for obesity. Here we review the most important aspects of irisin's action and discuss its involvement in energy and metabolic homeostasis and whether the beneficial effects of exercise in these disease states could be mediated by this protein. In addition the effects of irisin at the central nervous system (CNS) are highlighted. It is concluded that although current and upcoming research on irisin is very promising it is still necessary to deepen in several aspects in order to clarify its full potential as a meaningful drug target in human disease states.

## 1. Introduction

Obesity is at present the most common nutritional disease in industrialized countries, constituting a priority health problem. It is associated with the development of cardiovascular disease, *diabetes mellitus* type II, increased incidence of certain forms of cancer, and respiratory complications from other diseases, which leads to higher rates of mortality and morbidity, reducing directly or indirectly the quality and life expectancy of sufferers [1, 2]. Lifestyle modification, specifically changes in diet, physical activity, and exercise, currently continues to be the best option for treatment of obesity. In this sense, the benefits of exercise have been extensively documented [3]. Moreover, it has recently been reported that especially during or immediately after physical activity, skeletal muscle releases into circulation several hormones. These hormones, named myokines, can influence metabolism and modify cytokine production in different tissues and organs. On the basis of this, the concept of skeletal muscle must be reconsidered and being truly considered as an endocrine organ [4, 5].

Since human brown adipose tissue (BAT), especially in adults, was rediscovered several years ago by using positron emission tomography (PET) [6–9], it has been postulated as a major candidate for the treatment of obesity. This is based on the fact that brown adipose cells can dissipate energy in the form of heat leading to weight loss. This process takes place through a specialized mitochondrial protein called uncoupling protein 1 (UCP1). The uncoupling activity of UCP1 is explained by its ability to transport protons across the inner mitochondrial membrane, avoiding ATP synthesis and dissipating energy as heat [10]. Regulation of UCP1 is mainly at transcriptional level, where peroxisome proliferator-activated receptor *ϒ* coactivator 1*α* (PGC1*α*) plays a key role [11].

Studies in immortal preadipocyte lines from the brown adipose tissue of mice lacking PGC1*α* corroborated their importance in thermogenesis [12]. Another important characteristic is its role in mitochondrial biogenesis; in fact the increased expression of PGC1*α* is parallel to increased mitochondrial DNA and gene expression of OXPHOS system (oxidative phosphorylation) in BAT [13, 14]. Although PGC1*α* is mainly expressed in BAT, it is also expressed at higher levels in red, oxidative muscle. In fact, its expression is increased by exercise in mice, in rats, and in human beings [15]. Exercise rapidly and robustly increases the expression of PGC1*α*, but this effect is transient as both mRNA and protein levels of PGC1*α* quickly revert to preexercise values [16]. Exercise also activates AMP-activated protein kinase (AMPK), a master regulator of cellular metabolism. AMPK directly phosphorylates PGC1*α*, which is required for PGC1*α*-dependent induction of the PGC1*α* promoter [17]. While brief training produces only a transient rise in PGC1*α*, endurance training results in persistent PGC1*α* elevation [18]. Moreover, mice with transgenically increased PGC-1*α* in muscle showed improved metabolic responses as age related obesity and insulin insensitivity [19]. When the adipose tissue of these transgenic mice was analysed, it was observed that subcutaneous fat inguinal had significantly increased thermogenic gene program. These “brite” (brown-in-white) adipocytes display several classical brown adipocyte characteristics, as elevated levels of UCP1 mRNA and protein [20]. Further, other reports showed that exercise also enhances certain brown adipocyte-specific gene expression in the BAT, as well as white adipose tissue (WAT), suggesting that exercise training may induce important alteration in BAT and/or BAT-like phenotypic changes in WAT [21]. In this context it has been proposed that irisin, a recently discovered myokine, may be the molecule that links exercise with increased thermogenesis. In fact, irisin is named for Iris, the Greek goddess who served as courier among the Gods [20].

## 2. Irisin, A Bridge between Exercise and Thermogenesis

### 2.1. First Experimental Studies

**Figure 1:**
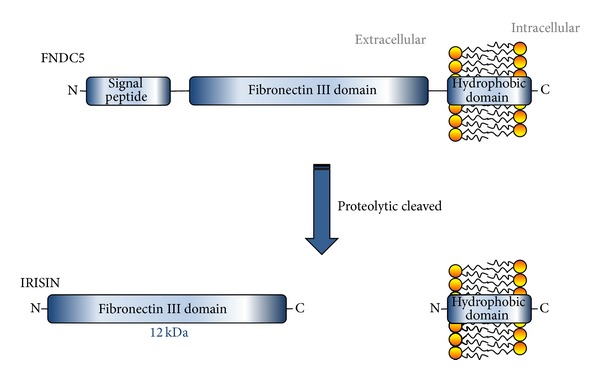
Expression of FNDC5 (fibronectin type III domain containing 5), also known as FRCP2 and PeP, is stimulated in muscle by PGC1-*α* in response to exercise. It is a signal peptide with two fibronectin domains in its amino (N)-terminal part and a hydrophobic domain inserted in the lipidic bilayer at carboxy (C)-terminal domain. The first 29 aa of the mouse FNDC5 are a signal peptide, followed immediately by the single FNIII domain of 94 aa. The next 28 aa are of unknown structure and function and contain the putative cleavage site for irisin. This is followed by a 19 aa transmembrane domain and a 39 aa cytoplasmic domain. FNDC5 is thus a type I transmembrane protein with its FNIII domain extracellular, similar to some cytokine receptors. This structure is synthesized as a type I membrane protein and followed by proteolytic cleavage realising amino (N)-terminal part of the protein into the extracellular to circulation.

Recently, Spielgman's group described that transgenic PGC-1*α* mice had greater levels of fibronectin type III domain containing (FNDC5) than wild-type mice [20]. FNDC5 (also known FRCP2 and PeP) is a type of transmembrane protein cloned by two groups in 2002. It has a signal peptide, two fibronectin domains and one hydrophobic domain inserted in the cell membrane [22, 23]. In fact, at present some authors question if FNDC5 might be a transmembrane receptor [24]. FNDC5 is proteolytically cleaved and secreted. Western blot of media fractions of cells overexpressing FNDC5 with antibodies against wild-type Fndc5 identified multiple bands; from 32 kDa to 20 kDa [20]. However, several aspects regarding proteolysis of this protein were not fully clarified yet. So, it seems that these possible discrepancies in molecular weight might be due to glycosylation in the culture media, while this is not observed in plasma mice. Moreover, the theoretically soluble secreted form, named irisin, would have a molecular weight of 12 kDa [20, 25] (Figure 1). A remarkable aspect about irisin is that the amino acid sequence is 100% identical among most mammalian species, which suggests a highly conserved function [20, 26].

 Boström and colleagues demonstrated that irisin has potent effects on the browning of certain white adipose tissues, both in culture and *in vivo*. So, when they applied FNDC5 to primary subcutaneous white adipocytes during differentiation a great increase in oxygen consumption was observed which suggests higher energy expenditure. Moreover, the increase in uncoupled respiration was accompanied by an important induction of UCP1 mRNA and other known brown fat genes. However, genes characteristic to WAT were downregulated. Surprisingly, FNDC5 showed almost no effects on the classical brown fat cells isolated from the interscapular depot [20].

 This evidence opened up some questions about the physiological role of irisin. In the same study,* in vivo*, it was demonstrated that injection intravenously of adenoviral vectors expressing full-length *Fndc5* resulted in *Ucp1* mRNA increased in the subcutaneous depot. Moreover, a moderate increase irisin blood levels caused a significant improvement in energy expenditure, body weight, and insulin resistance in mice that were fed a high fat diet. Finally, it was demonstrated that irisin was required for the effect of exercise in the browning of subcutaneous white fat and concluded that the rise in irisin is mediated by augmented concentrations of PGC1*α* in muscle, while PPAR-*α* (peroxisome proliferator-activated receptor-*α*) acts as downstream target of this hormone [20]. 

### 2.2. Interplay with Other Myokines

There is an extensive literature about different exercise-related signals that can regulate the expression and/or secretion of the diverse myokines [4, 5, 27, 28]. In this context, it has recently been published that there is a close interaction between irisin and myostatin. Myostatin, besides being a critical autocrine/paracrine inhibitor of skeletal muscle growth, has been shown to play an important role in metabolism [29]. In fact, it has been described as myostatin-knockout mice (Mstn^−/−^) that show an increase muscle mass and a concomitant reduction of fat mass. Moreover, these mice show WAT with characteristics of BAT, an effect mediated by the AMPK-PGC1*α*-FNDC5 pathway in muscle [30].

Other studies have tried to elucidate the irisin role and other myokines in different physiological conditions. When male rats were subjected to calorie restriction (CR; ~60% *ad libitum*) there were no significant diet-related differences in plasma levels of myonectin, myostatin, or irisin, although there were significant changes in fat and lean mass, and also in insulin resistance [31]. These results may indicate that alterations in plasma concentration of these proteins are not essential for the CR-related improvement in insulin sensitivity in rats; however, it does not rule out that these plasma proteins may be relevant for some of caloric restriction's metabolic effects. On the other hand, Sánchez and collaborators have studied the possible effects of free fatty acids (FFA) alone and combined with adrenaline and AICAR (an activator of AMPK that acts as an exercise mimetic precursor) in the production of the myokines IL6, IL15, and irisin in mouse muscle cells *in vitro* [32]. They observed that FFA, adrenaline, and AICAR have a great influence in the IL6 expression and secretion, a little inhibitory effect on IL15 expression and almost no effect on the expression of FNDC5. In fact, the authors only found that FNDC5 had a tendency to be reduced with FFA and AICAR at isolated specific time points. Thus, it would be possible that more signals may be required *in vivo* for inducing FNDC5 expression. In this sense, recent evidence using human rhabdomyosarcoma cells showed that treatment for 24 and 48 hours with omega 3 fatty acids significantly induced irisin expression [33]. Finally, it has also been found that just as FNDC5, heart-derived natriuretic peptides activate white adipose thermogenic programs [34]. Taken together, these results may suggest that tissues such as skeletal and heart muscle, involved in high energy-expending activity, send signals to adipose tissue [35, 36].

### 2.3. Irisin Is Also an Adipokine

Current data by Roca-Rivada and coworkers proposed that irisin is not only secreted by muscle tissue. In fact, they demonstrated that irisin is a new adipokine with an important autocrine and endocrine function. Moreover, they showed that FNDC5/irisin has a different pattern of secretion depending on the anatomical location of adipose tissue. Thus, subcutaneous adipose tissue secretes more much FNDC5/irisin than visceral adipose tissue, reflecting one more time that visceral fat is more implicated in metabolic complications, while subcutaneous fat has a possible beneficial role. They also showed that short-term periods of exercise training induced FNDC5 secretion by WAT, that this secretion was significantly reduced in fasting animals, and that WAT of obese animals had an increase secretion of this hormone suggesting a type of resistance [25]. Another interesting feature, also reported by those authors, indicates that FNDC5/irisin has a secretion profile similar to other adipokines like leptin. Moreover, it is suggested that this hormone might be implicated in the regulation of circulating FNDC5/irisin levels. In fact, Zucker obese rats with no functional leptin receptor showed significantly diminished levels, while DIO (diet induce obesity) rats showed a significant increase. Ultimately, all these results suggest an interactions between muscle and adipose tissue interaction a regulatory feedback mechanism.

In this same context, Roberts et al. showed that obese/diabetic prone Otsuka Long-Evans Tokushima Fatty (OLETF) rats have more muscle expression FNDC5 than lean Long Evans Tokushima Otsuka (LETO); however, LETO rats have higher circulating irisin levels. The authors also observed that triceps FNDC5 mRNA expression was correlated with fat mass and with plasma leptin; however, *in vitro* leptin treatment had no effect on FNDC5 mRNA expression in myotubes [37]. Given that the effect of leptin treatment depends on endogenous levels of this hormone and on the physiological state [38], many studies are still needed to determinate a possible interaction between leptin and irisin in the so-called muscle-adipose tissue axis. 

## 3. Irisin, Potential Roles in the Central Nervous System

 Besides interaction between skeletal muscle and adipose tissue, it has been described that FNDC5/irisin might have a role in the central nervous system. In fact, it has already described previously that PGC1-*α*, an upstream of FNDC5, benefits tissues that do not have a primary metabolic function, such as the brain [39–41]. In this context, immunohistochemical studies have recently revealed that rat and mice cerebellar Purkinje cells expressed irisin and also FNDC5 [42]. Furthermore, the same authors hypothesize about a novel neural pathway, where irisin produced in cerebellum might regulate adipocyte metabolism via several intermediary synapses in the medulla and spinal cord, an interesting idea that still requires to be confirmed.

 Supporting the role of FNDC5/irisin in the nervous system, it should be noted another study where it is demonstrated that FNDC5 is required for the adequate neural differentiation of mouse embryonic stem cells (mESCs) [43]. The authors observed that both *Fndc5 *knockdowns in mESCs during their differentiation after postneuronal progenitor formation and the neuronal differentiation were reduced. Finally, Moon et al. showed that hippocampal neurogenesis is regulated by irisin in a dose-dependent manner. So, while physiological concentrations of irisin (5–10 nmol/L) had no effect on mouse H19-7 hippocampal neuronal cells proliferation, pharmacological concentrations (50–100 nmol/L) increased proliferation when they were compared to control. This increase seems to occur trough signal transducer and activator of transcription (STAT)3 but not AMPK and/or extracellular signal-regulated kinase (ERK) signalling pathways [44].

 Overall this evidence suggests a central role for irisin, In this regard, considering that the hippocampus is one of the principal regions affected by Alzheimer's disease and that exercise causes neurogenesis in humans reducing risk of Alzheimer's [45], Parkinson's, and some other neurodegenerative diseases [26, 46], irisin might be the link between exercise and healthy brain. Another interesting question that needs to be addressed is whether irisin may be expressed and play a role in other brain areas involved in the regulation of energy balance, such as the hypothalamus and the brainstem.

## 4. Irisin, Studies in Humans

### 4.1. Human Exercise Gene

As stated above, irisin has a highly conserved function, and as in rodents, in humans this hormone is also predominantly expressed in muscle [47]. While, available data indicates that this is the main source of production, it was also found that both subcutaneous and visceral adipose tissue expressed and secreted FNDC5/irisin [25, 48]. On other hand, circulating irisin was detected in the serum or plasma of all subjects studied, whereas circulating FNDC5 was detected in only a minority of the subjects [47], which could be explained by a different processing in a minority group of humans.

Throughout the past two years, several studies in humans have tried to clarify the role of FNDC5/irisin in physiological conditions and in disease states. Spielgman's group showed that endurance exercise training for 10 weeks in healthy adult humans increased plasma irisin levels compared to the baseline state [20]; however, there are some discrepancies about this. While Huh et al. also observed that circulating irisin levels were significantly upregulated 30 min after acute exercise [47], another study have questioned those results. So, other study has not been able to reproduce FNDC5 gene activation by aerobic exercise in younger subjects or in a resistance training study in 20–80 year olds [49]. These authors question therefore whether irisin is a human exercise gene. These discrepancies might be explained as that an increase in irisin levels occurs in states where more energy is needed, such as untrained individuals, while among trained individuals it is not necessary [47]. In the same direction, a recent study confirms that neither longer-term nor single exercise markedly increases skeletal muscle FNDC5 expression or serum irisin [50].

It seems, hence, that exercise might have an effect on irisin levels depending on physiological condition. In this sense, a new study describes that patients subjected to hemodialysis seem to have lower plasma irisin than healthy subjects and also show exercise training resistance; so despite increasing muscle mass, they not have higher irisin levels [51].

### 4.2. Metabolic Diseases

When analyzing the correlation between body max index (BMI) and irisin levels, differences were also found. Some studies observed a positive correlation with BMI [47, 52, 53], while other reported null [49] or even a negative correlation [48]. It would be necessary a deeper investigation in this field, and a possible resistance to this protein should be characterized, as animal studies suggest [25]. In addition, bariatric surgery-induced weight loss has been reported to decrease irisin levels, independently of BMI [47]. However the functional significance of this finding needs further exploring.

Similarly, it has been established by some groups a relation between *diabetes mellitus* type 2 (DMT2) and irisin levels, although it is also reported that irisin expression is not related to diabetes status in humans [49]. Most studies show lower irisin levels in patients with DMT2 [48, 54, 55]. Fernandez-Real's group suggests that a lesser production of irisin in muscle/adipose tissue in obese and patients with DMT2 could be responsible of the obesity-associated lower brown or beige adipocytes in human adipose tissue. So, they consider increasing irisin levels and browning adipose tissue as a potential target for metabolic diseases' treatment [48].

In this same context, another controversy has been reported. The study of single nucleotide polymorphisms (SNPs) in the human *Fndc5* locus, encoding the irisin precursor, showed that a common genetic variation in this locus determines insulin sensitivity [56]. Moreover, data from human myotubes revealed a negative association between FNDC5 expression and *in vivo* measures of insulin sensitivity. This result appears conflicting with the mouse data from Boström et al. who reported reduced insulin resistance in high fat-fed mice after adenoviral *Fndc5* overexpression [20]. Considering the association of DMT2 and cardiovascular disease, a role for irisin is also tempting to be speculated. In this sense the FNDC5 expression in a skeletal muscle biopsy from heart failure (HF) patients, it was observed that this expression relates to functional capacity in a human HF and that a decrease in FNDC5 expression might reduce aerobic performance in HF patients [57].

Circulating irisin has been also found to be directly associated with muscle mass and estradiol levels and inversely associated with age in middle-aged women. Also it is negatively correlated with age, insulin, cholesterol, and adiponectin levels [47, 52, 58], as well as intrahepatic triglyceride contents in obese adults [59]. While, another paper suggests that in a population of postmenopausal women with BMI between 24 and 45, irisin levels do not correlate with 24 h energy expenditure (EE); however, for a subpopulation with EE greater than predicted, irisin levels and EE are highly correlative [60]. 

Similar to physical activity, drugs might also increase irisin levels and thus affect lipid metabolism and improve risk among dyslipidemic and/or obesity individuals. Given recent data, everything seems to indicate that between these drugs, statins could have an important role in this sense [61]. In this context, recently, Gouni-Berthold and collaborators have described that simvastatin, a hypolipidemic drug member of the statins, increases irisin concentrations both *in vivo* as *in vitro* [62]. Although it could be postulated that this increase could be beneficial, for example, by influencing adipose tissue metabolism and insulin resistance, it will be necessary to determine if irisin levels are result of myocyte damage or/and a mechanism of statin-induced cellular stress protection [62].

 Another disease with altered energy expenditure and with high prevalence of metabolic imbalance and abnormal energy homeostasis is also chronic kidney disease (CKD). It was observed that patients with CKD have lower irisin levels at rest, independently of high-density lipoprotein cholesterol levels. The mechanism underlying the decrease in irisin in CKD is unknown, even though it seems that indoxyl sulphate, which is a protein-bound uremic toxin, decreases FNDC5 expression in skeletal muscle cells and irisin level in the cell culture medium [58]. Authors consider that these results show good evidence on how uremia may affect irisin levels. Although this study has some limitations, it is suggested that irisin may be a novel therapeutic agent for treating metabolic diseases in CKD patients.

## 5. Future Prospects

 When Böstrom and colleagues described irisin, rapidly, it was seen its great therapeutic potential. Irisin was seen as possible treatment for diabetes and perhaps also therapy for obesity. Moreover, it also was considered a possibility to treat patients with Alzheimer's, Parkinson's, and some other neurodegenerative diseases. However, new studies have started to question the initial expectations [24, 63]. So, while clear-cut data have been reported in rodents, the thermogenic effect of irisin in humans remains controversial, and it is not clear if exercise has an impact on irisin levels [49]. In fact, recently, Raschke and coworkers described that neither FNDC5 gene is activated by contraction in humans nor has effect on “brite” differentiation of human preadipocytes [63]; even they propose that irisin function for mice is lost in humans. Thus, it seems obvious that further studies are needed to elucidate, in depth, this field.

 First more studies would be necessary to determinate what the precise role of different forms of FNDC5/irisin is and if there is a different mechanism of proteolysis as it already was suggested [47]. On the other hand, it is absolutely necessary to characterize the receptor and the signalling pathway, which will allow a better understanding of irisin function. Just as with other hormones it seems to be a tolerance or resistance mechanism to irisin [25, 60]. So, the factors that contribute to irisin tolerance and/or resistance also would be defined. Similarly more extensive studies, with different cohorts, assessing genetic variations in the irisin gene and its relationships to obesity and associated comorbidities across life span are eagerly awaited. Another important aspect that we need to consider is that human BAT is closely related to rodent beige fat, rather than classical BAT; so if we want to study the irisin effect in human BAT, a rodent model with beige fat would be necessary [26]. Intensive research efforts are needed to use BAT as a target organ for treatment of metabolic diseases.

**Figure 2:**
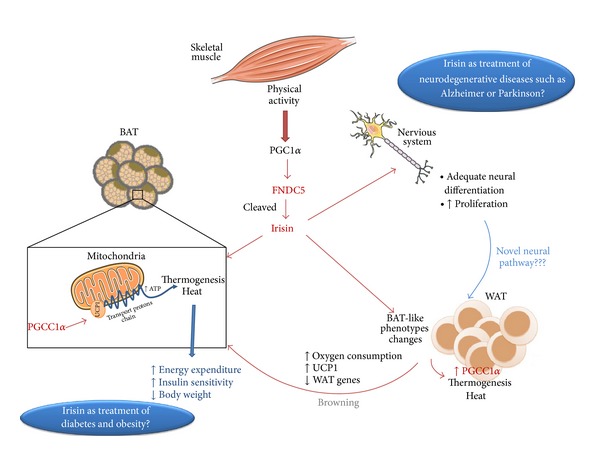
Skeletal muscle releases to circulation several hormones denomined myokines acting as endocrine organ. Thus during exercise PGC1*α* is activated inducing FNDC5 release which is cleaved to irisin. Irisin can act on different tissues, thereby brown adipose tissue activates UCP1 in mitochondria triggering transport protons chain in the mitochondrial membrane, resulting ATP increased and dissipating energy in form of heat. This process increases energy expenditure, reduces body weight, and improves metabolic parameters such as insulin sensitivity. Irisin on white adipose tissue stimulates BAT-like phenotypes changes, increasing PGC1*α* expression and thereby UCP1 and oxygen consumption while decreases WAT genes, process in which WAT stops behaving as energy reservoir for to use fat as energy source as in BAT, process named browning. For all of this, irisin has been proposed as a possible novel treatment in diabetes and obesity. Other target of irisin is nervous system where preliminary studies suggest that it could act on adipocyte metabolism through a novel neural pathway and on the other hand irisin induces neural proliferation and adequate neural differentiation, so it could also be a therapeutic target for neurodegenerative diseases such as Alzheimer or Parkinson.

In conclusion, although current and upcoming research on irisin is very promising and nowadays we already know so much about it (Figure 2), it is still necessary to deepen in several aspects in order to clarify its full potential as a meaningful drug target in human disease states.
